# Supplementary material for: Spatial (mis)match between EEG and fMRI signal patterns revealed by spatio-spectral source-space EEG decomposition
Source: Front Neurosci. 2025 Mar 14;19:1549172. doi: 10.3389/fnins.2025.1549172 (PMC11949981; doi:10.3389/fnins.2025.1549172)
Supplement: Supplementary file 1 [file Image_1.pdf]

Supplementary Material

1 SUPPLEMENTARY FIGURES

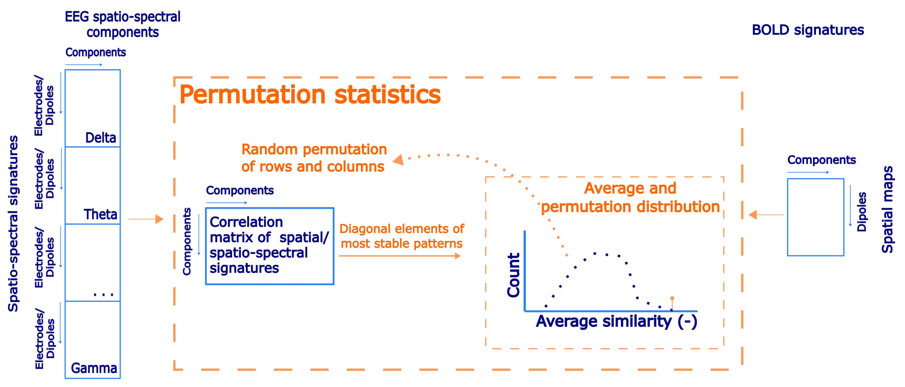

Figure S1. A schematic flowchart of a permutation spatial statistical testing of EEG spatio-spectral signatures and statistical GLM maps.

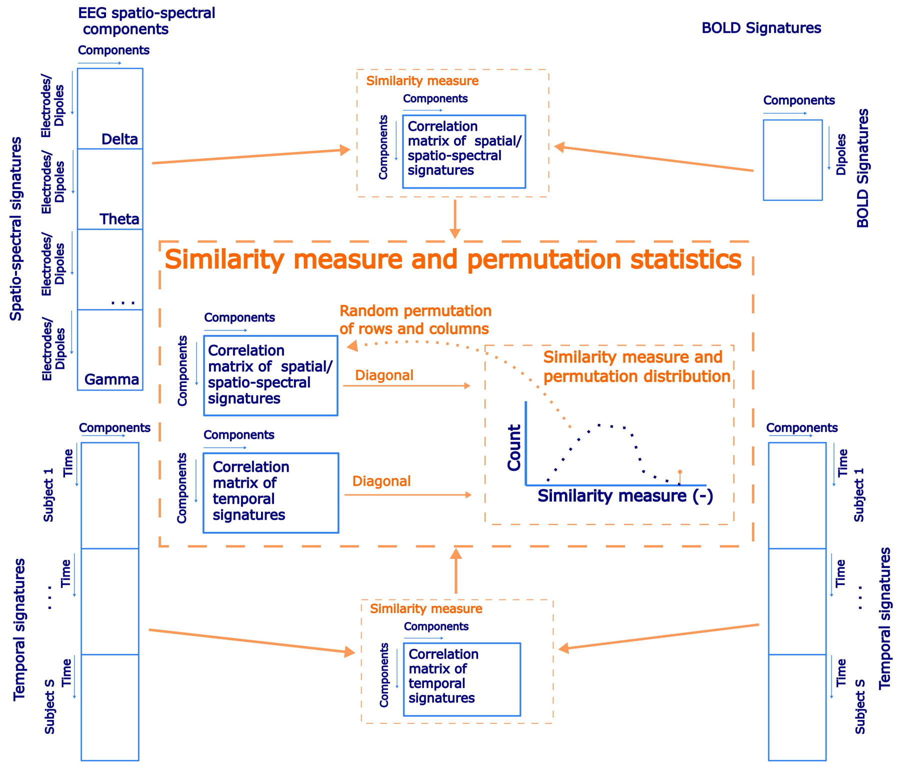

Figure S2. A schematic flowchart of a permutation spatio-temporal statistical testing of EEG spatio-spectral signatures and statistical GLM maps.

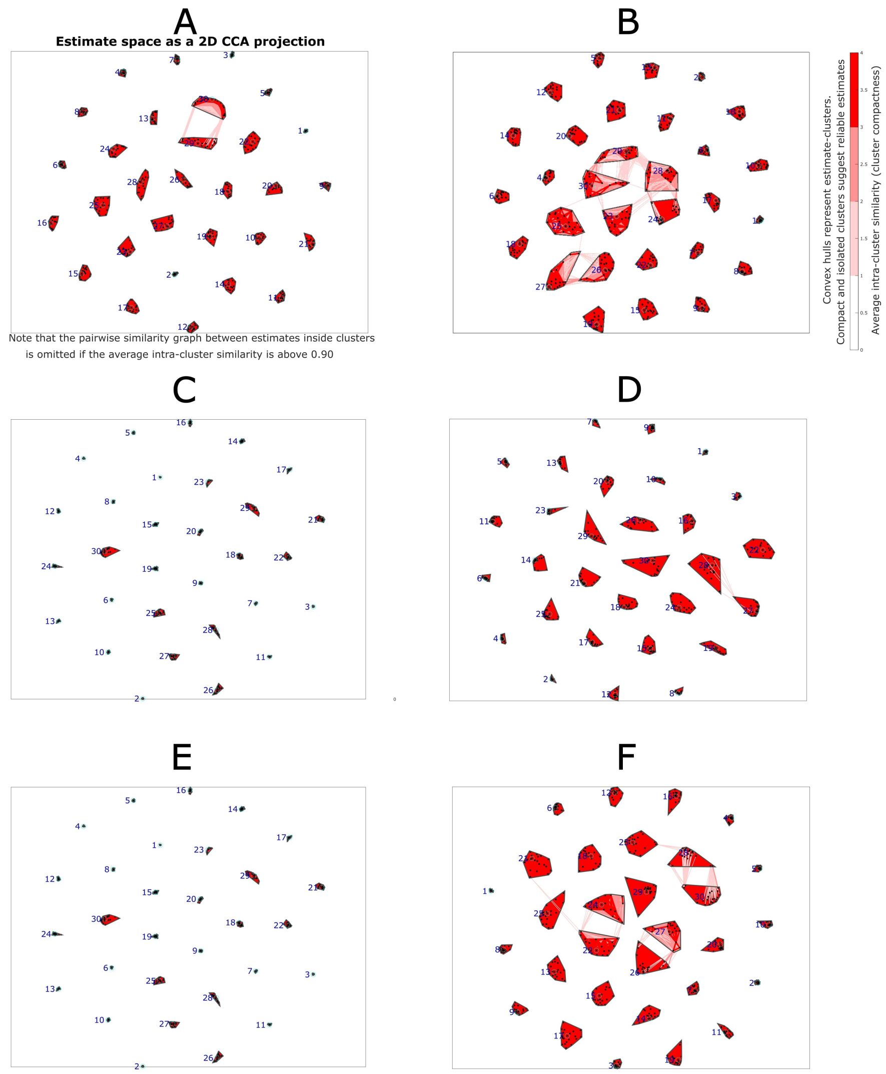

Figure S3. ICASSO visualization of defined clusters as 2D CCA projection for: **(A)** whole data set, electrode-space, **(B)** whole data set, source-space, **(C)** subset 1, electrode-space, **(D)** subset 1, source-space, **(E)** subset 2, electrode-space, **(F)** subset 2, source-space.

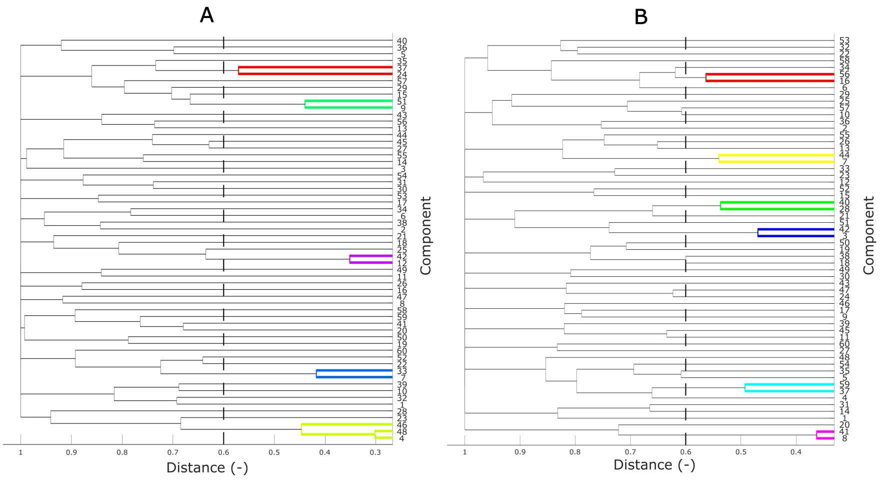

Figure S4. Hierarchical clustering dendrogram of components from both subsets labeled as 1-30 for components from the first subset and 31-60 as components from the second subset with nodes definition by a shortest distance method. The distance metric between components is defined by Equation 4 in the main text. Dashed line denotes a distance cut-off to define clusters both in **(A)** electrode-space, and **(B)** source-space spatio-spectral decompositon.
